# Aspherical Lens Design Using Genetic Algorithm for Reducing Aberrations in Multifocal Artificial Intraocular Lens

**DOI:** 10.3390/ma8095305

**Published:** 2015-09-17

**Authors:** Chih-Ta Yen, Shih-Cyuan Jin

**Affiliations:** Department of Electrical Engineering, National Formosa University, Yunlin 632, Taiwan; E-Mail: rupg4fm06@gmail.com

**Keywords:** intraocular lens, third-order aberrations, anterior lens, posterior lens, genetic algorithm, optimization

## Abstract

A complex intraocular lens (IOL) design involving numerous uncertain variables is proposed. We integrated a genetic algorithm (GA) with the commercial optical design software of (CODE V) to design a multifocal IOL for the human eye. We mainly used an aspherical lens in the initial state to the crystalline type; therefore, we used the internal human eye model in the software. The proposed optimized algorithm employs a GA method for optimally simulating the focusing function of the human eye; in this method, the thickness and curvature of the anterior lens and the posterior part of the IOL were varied. A comparison of the proposed GA-designed IOLs and those designed using a CODE V built-in optimal algorithm for 550 degrees myopia and 175 degrees astigmatism conditions of the human eye for pupil size 6 mm showed that the proposed IOL design improved the spot size of root mean square (RMS), tangential coma (TCO) and modulation transfer function (MTF) at a spatial frequency of 30 with a pupil size of 6 mm by approximately 17%, 43% and 35%, respectively. However, the worst performance of spherical aberration (SA) was lower than 46%, because the optical design involves a tradeoff between all aberrations. Compared with the traditional CODE V built-in optimal scheme, the proposed IOL design can efficiently improve the critical parameters, namely TCO, RMS, and MTF.

## 1. Introduction

Spherical aberration is a naturally occurring disorder of the human eye. Eye curvature is not the spherical diameter equidistant; careful observation of the cornea and lens curvature of the more prominent central part of the eye, which is surrounded by a relatively flat section, this way, the light entering the eye elapses because the refraction angle will exhibit a degree of aberration [1,2,3,4,5].

The main objective of this study was to devise a method to design intraocular lenses (IOLs) for correcting myopia and astigmatism. Eyes are the most important information source for humans. The crystalline lens will be overused and will not be able to focus if people stare at a nearby object for a long time, which will eventually cause myopia. With the development of consumer electronics products nowadays, people carry them all the time. Everyone has a cell phone or tablet, which constantly exposes people to blue light. As a result, more and more young people have problems with their macula luteas, causing blurred vision. The phacoscotasmus caused by macular degeneration is one of the major reasons for myopia and even blindness [6]. Apart from 3C products, some congenital symptoms can cause light to focus in advance because of the overlong axis oculi [7,8,9] and also myopia. Although this can be corrected with glasses, artificial crystalline lenses have become an important option for critical patients. For the improvement of these symptoms, there have been many ongoing studies on crystalline lenses in human eyes. However, crystalline lenses are very difficult to correct, as they are inside human eyes. Hence, the technology of artificial crystalline lenses was proposed. Artificial crystalline lenses are of great help to people with severe myopia as well as cataracts and glaucoma [10]. The older a person is, the easier it is to lose the original flexibility of the crystalline lens. This loss of flexibility results from the accumulation of protein and produces symptoms of presbyopia. The general treatment prescribed is the use of presbyopic glasses and glasses. However, extra plus lenses inconvenience people. Therefore, researchers began exploring ways to improve human vision without using glasses. The corneal or crystalline lens is located in the interior of the human eye and is therefore difficult to correct.

In general, most commercial optical design software using damped least square (DLS) optimization [11] is best for miniaturizing the spot size. The reference [12] discussed about the commercial optical design software of (CODE V) built-in DLS optimization with genetic algorithm. In the research, it concluded that in all run, the genetic algorithm (GA) optimization was better than the classical DLS optimization. Because the stochastic nature of GA optimization gain obtained in it varies from one run to another. Optical system designers must sometimes depend on experience when they try to balance these aberrations. Therefore, optimizated algorithm such as GA is introduced to optimize the optical performance with optical design software. There is some research discussed about the GA with optical design software. It used to suppress primary chromatic aberration in an advanced telephoto lens [13]. Miniature lens design and optimization with liquid lens element via genetic algorithm [14]. Extended optimization of chromatic aberrations via a hybrid Taguchi–genetic algorithm for zoom optics with a diffractive optical element [15]. Lens design use real-coded genetic algorithms for global and multi-objective optimization [16].

In the present study, a method was devised for designing a suitable IOL for different conditions of the human eye. First, we utilize the CODE V internal human eye model to simulate an imperfect human eye and use an optical design scheme involving an aspherical lens for designing the IOL. Second, a genetic optimization algorithm is used to achieve the best results for human image qualities. The optimization principles of genes are mainly about the application selection, copulation, and mutation, and the optimized image quality is obtained by calculating the optimal solution to the parameters of the lens group of the optical system. Because changing the corneal or crystalline lens in the human eye is difficult, using a well-designed artificial IOL for imperfect human eyes is crucial. Hence, an IOL design with good imaging performance can eliminate the use of external lenses for correcting vision. The rest of this paper is organized as follows. This study introduces the condition of myopia at an early stage, but of course, the proposed method can also be applied to hyperopia, presbyopia, *etc.* The optimal image quality for human eyes is obtained by the optimization of the artificial crystalline lens via the optical design software CODE V and genetic algorithms from the viewpoints of optical system designs. Accurate focus can be achieved, even without exterior eyeglasses. Section 2 provides an overview of the methodology of the optical IOL design. In Section 3, we analyze the theory and formulas and discuss the proposed simulation performance. Section 4 summarizes the conclusions.

## 2. Methodology of Optical IOL Design

This study used an aspherical technique and a genetic algorithm (GA) to design an artificial IOL for reducing aberrations in an imperfect human eye. The design consisted of two steps: (1) We used the internal human eye model of Liou and Brennan [17] in CODE V to model the human eye lens patterns among basis. In the aspherical technique used for simulating a human eye IOL, parameters such as the aspherical cone coefficient, curvature, and thickness are adjusted to modify the original surface of the IOL for adjusting the focal length of the objective. (2) The GA scheme was used for optimizing the section, for reducing aberrations and improving the modulation transfer function (MTF). It enables GA optimization to be performed in a manner that minimizes the error function, thereby helping achieve more accurate focusing.

## 3. Theory and Formula Analysis

### 3.1. Aspherical Lens

**Figure 1:**
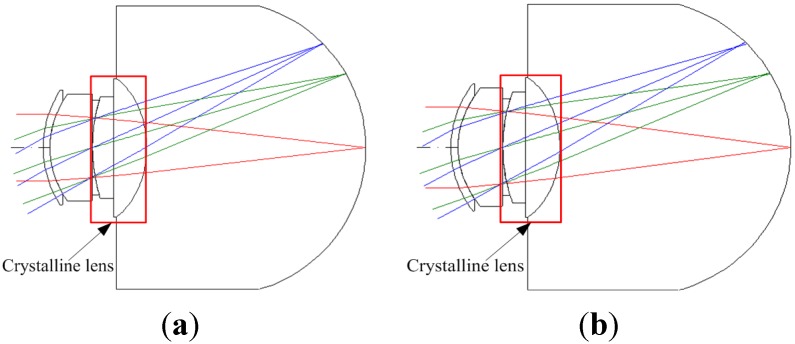
Two-dimensional (2D) layout diagram of the initial crystalline lens [17]. (**a**) 5 mm; (**b**) 6 mm.

We used the internal optical human eye model in CODE V, which was proposed by Liou and Brennan in 1997. However, this model is mainly based on the nonspherical development; therefore, for designing an IOL, changing its aspherical curvature and thickness was essential. Table 1 lists the initial specifications of the crystalline lens of the human eye in the IOL design. The human eye crystalline lens two-dimensional (2D) layout is show in Figure 1.

**Table 1:** Initial specifications of the crystalline lens of the human eye.

Initial Conditions of Design	
Anterior radius of the crystalline lens	0 mm
Posterior radius of the crystalline lens	0.0806 mm
Anterior thickness of the crystalline lens	1.59 mm
Posterior thickness of the crystalline lens	2.43 mm

The effect of higher-order aberrations such as spherical aberration (SA) and tangential coma (TCO) on human vision depends mainly on the size of the pupil. Usually, it is about 5–6 mm around the pupil size of the human eye. Pupil size is larger at night than during the day because of more nearly the amount of incident light is needed in the dark environment. Large pupil size induces large amount of incident light into eye, it will therefore produce glare effect and enlarge spot size. The research currently takes the entrance pupil of 5 mm and 6 mm to analyze the effects in different pupil sizes of human eye.

The spot size is the root mean square (RMS) radius of the faculae’s radial dimension. By first obtaining the square distance between each light ray and the reference point, the mean value of all light rays can be obtained. Finally, the RMS is calculated as the distance between the section ($x_{i}y_{i}$) and the reference point ($x_{0}y_{0}$) of each critical light ray on the image plane. The formula for calculating the RMS is as follows:

(1) $$R_{\text{RMS}}=\sum^{\text{​}}\sqrt{\left[{\left(x_{i}-x_{0}\right)}^{2}+{\left(y_{i}-y_{0}\right)}^{2}\right]/n}$$

In the formula, *n* is the total number of light rays. A higher number of light rays results in a more accurate RMS radius. If all faculae are situated within the airy disk, the light energy is more centralized, and this optical system is close to the diffraction limit. Table 2 presents the third-order aberrations and RMS of the initial human eye model.

**Table 2:** Third-order aberrations in the initial human eye model. SA: spherical aberration; TCO: tangential coma; RMS: root mean square.

	Aberration	SA	TCO	RMS
Pupil Size				
5 mm		−0.016148	−0.033068	0.005315
6 mm		−0.027904	−0.047618	0.01264

In this study, in the aspherical mathematical description, we neglected the higher-order aspherical coefficients; the formula can be written in the conic form presented in Equation (2). Thus, learned addition aspherical coefficient, which can change the radius of curvature and obtain the results of aspherical patterns. In this program, we modified the curvature of the lens to improve human eye lesions.

(2) $$Z=\frac{X^{2}}{R+\sqrt{R^{2}-(1+K)X^{2}}}$$

Here, *Z* represents lens sag (*i.e.*, the rotational symmetry axis of aspherical), *R* is the radius of curvature of the vertex, *X* denotes the rotational symmetry axis of aspherical, and *K* represents the conic coefficient.

**Figure 2:**
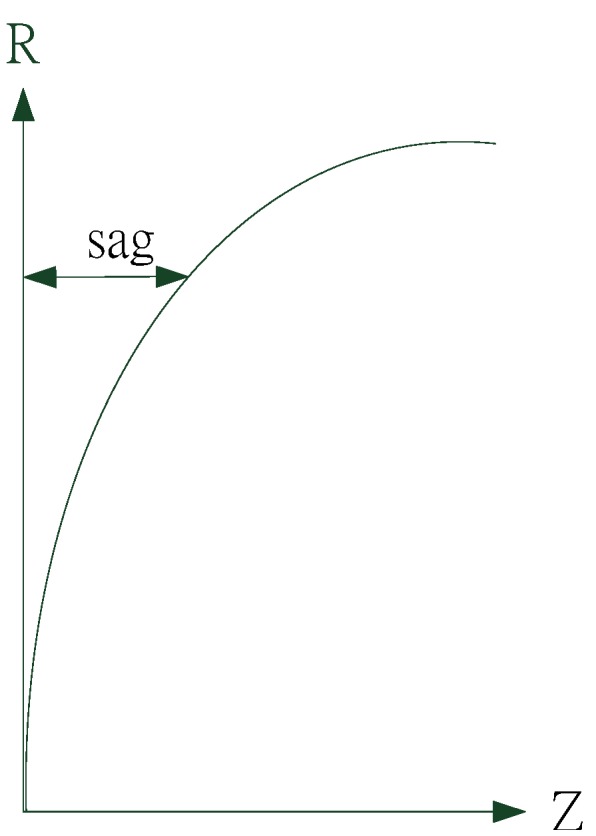
Schematic of aspherical.

**Figure 3:**
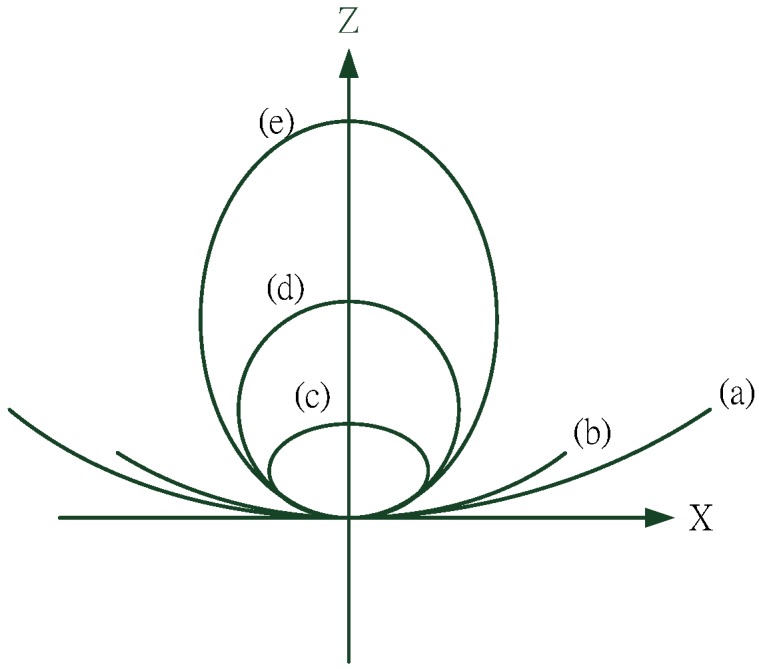
Conic curve. (**a**) *K* < −1; (**b**) *K* = 1; (**c**) *K* > 1; (**d**) *K* = 0; (**e**) −1 < *K* < 0.

Figure 2 shows a schematic of the aspherical, and Figure 3 presents a schematic of the conic section.

**Figure 4:**
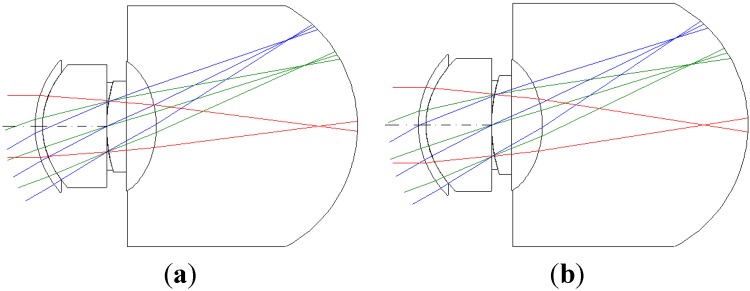
The 2D layout illustrating 550 degrees myopia and 175 degrees astigmatism. (**a**) 5 mm; (**b**) 6 mm.

We chose polymethyl methacrylate as the IOL material. This study is expected to assist optical designers in further optimizing the IOL (in this study, myopia is 550 degrees myopia and 175 degrees astigmatism) after normal optimization by using current optical design software for achieving the theoretical maximum performance. For optical design purposes, we mainly calculated the parameters of the myopic and astigmatic states by first calculating the corneal lens and then the lens of the humor, as shown in Figure 4.

The myopic and astigmatic effects are calculated using Equations (3) and (4) [18]:

(*N*/100) × 0.1 = cornea (3)

[(*M* + (*N*/2)]/100 × 0.37 = humor (4)

where *M* and *N* are parameters related to myopia and astigmatism, respectively. We then calculated the design parameters of the corneal surface and the surface of the humor to express the states of myopia and astigmatism. Table 3 reports the third-order aberrations of an imperfect human eye with myopia and astigmatism.

**Table 3:** Third-order aberrations for 550 degrees myopia and 175 degrees astigmatism.

	Aberration	SA	TCO	RMS
Pupil Size				
5 mm		−0.073302	−0.052464	0.537989
6 mm		−0.126666	−0.075548	0.698584

**Figure 5:**
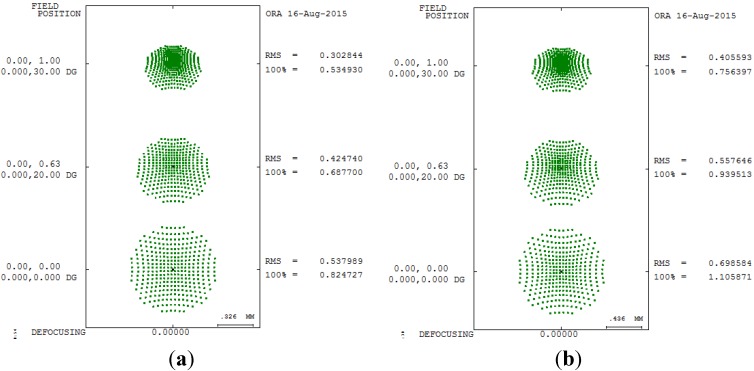
Spot size diagram of 550 degrees myopia and 175 degrees astigmatism. (**a**) 5 mm; (**b**) 6 mm.

The distribution of energy can be determined through spot size analysis of the focus point, and the spot size is mainly determined using the radial root mean square. Figure 5 shows the spot size analysis of 550 degrees myopia and 175 degrees astigmatism in different pupil sizes.

**Figure 6:**
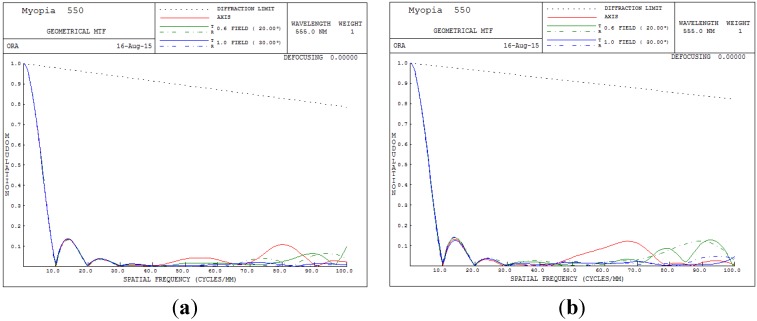
Modulation transfer function (MTF) plot for 550 degrees myopia and 175 degrees astigmatism. (**a**) 5 mm; (**b**) 6 mm.

The overall clarity of images in the optical system can be judged from the MTF performance. The higher the MTF value for all spatial frequencies, the higher is the achievable image performance. Figure 6 presents the MTF plot for the human eye conditions of 550 degrees myopia and 175 degrees astigmatism. In Figure 6, the image quality clearly becomes unacceptable when the spatial frequency exceeds 8 cycles/mm.

The lens parameters of Figure 4 after CODE V and GA optimizations are listed in Table 4, Table 5, Table 6 and Table 7 for different pupil sizes, respectively. The surface numbers listed in Table 4, Table 5, Table 6 and Table 7 are according to 2D layout of Figure 4. The surface number (surface #) listed in Table 4, Table 5, Table 6 and Table 7 are according to 2D layout of Figure 4 of each lens location from left to right, for example, the surface #Object is the object plane of Figure 4, the surface #1 is the first lens of Figure 4, the surface #2 is the second lens of Figure 4, the surface #Stop is the third lens of Figure 4, the surface #4 is the fourth lens of Figure 4, *etc*. and the surface #Image is the last lens of Figure 4.

**Table 4:** The lens parameter after CODE V optimization for pupil size 5 mm.

Surface#	Surface Type	Y Radius	Thickness	Glass	Refract Mode	Y Semi-Aperture
#Object	Sphere	0	Infinity	-	Refract	-
#1	Conic	0.1287	0.6	“cornea”	Refract	4.6712
#2	Conic	0.1563	5.195	“humor”	Refract	4.3066
#Stop	Sphere	0	0	“humor”	Refract	3
#4	Conic	0.0707	0.0915	ACRYLIC_SPECIAL	Refract	2.0413
#5	Sphere	0	1.2623	ACRYLIC_SPECIAL	Refract	2.2640
#6	Conic	−0.0853	16.27	“humor”	Refract	2.6105
#Image	Sphere	−0.0909	0	“humor”	Refract	7.7009

Note: Designed conic coefficient (Surface #4 is −24.88; Surface #6 is 1.37).

**Table 5:** The lens parameter after proposed genetic algorithm (GA) optimization for pupil size 5 mm.

Surface#	Surface Type	Y Radius	Thickness	Glass	Refract Mode	Y Semi-Aperture
#Object	Sphere	0	Infinity	-	Refract	-
#1	Conic	0.1287	0.6	“cornea”	Refract	4.6712
#2	Conic	0.1563	5.195	“humor”	Refract	4.3066
#Stop	Sphere	0	0	“humor”	Refract	3
#4	Conic	0.0755	0.9283	ACRYLIC_SPECIAL	Refract	2.0408
#5	Sphere	0	1.0016	ACRYLIC_SPECIAL	Refract	2.2688
#6	Conic	−0.0849	16.27	“humor”	Refract	2.5282
#Image	Sphere	−0.0909	0	“humor”	Refract	7.6499

Note: Designed conic coefficient (Surface #4 is −27.2; Surface #6 is 1.69).

**Table 6:** The lens parameter after CODE V optimization for pupil size 6 mm.

Surface#	Surface Type	Y Radius	Thickness	Glass	Refract Mode	Y Semi-Aperture
#Object	Sphere	0	Infinity	-	Refract	-
#1	Conic	0.1287	0.6	“cornea”	Refract	4.6712
#2	Conic	0.1563	5.195	“humor”	Refract	4.3066
#Stop	Sphere	0	0	“humor”	Refract	3
#4	Conic	0.0819	0.9763	ACRYLIC_SPECIAL	Refract	2.0413
#5	Sphere	0	1.2627	ACRYLIC_SPECIAL	Refract	2.2640
#6	Conic	−0.0702	16.27	“humor”	Refract	2.6105
#Image	Sphere	−0.0909	0	“humor”	Refract	7.7009

Note: Designed conic coefficient (Surface #4 is −11.24; Surface #6 is −3.85).

**Table 7:** The lens parameter after proposed GA optimization for pupil size 6 mm.

Surface#	Surface Type	Y Radius	Thickness	Glass	Refract Mode	Y Semi-Aperture
#Object	Sphere	0	Infinity	-	Refract	-
#1	Conic	0.1287	0.6	“cornea”	Refract	4.6712
#2	Conic	0.1563	5.195	“humor”	Refract	4.3066
#Stop	Sphere	0	0	“humor”	Refract	3
#4	Conic	0.0791	1.1739	ACRYLIC_SPECIAL	Refract	2.0408
#5	Sphere	0	1.1021	ACRYLIC_SPECIAL	Refract	2.2688
#6	Conic	−0.0723	16.27	“humor”	Refract	2.5282
#Image	Sphere	−0.0909	0	“humor”	Refract	7.6499

Note: Designed conic coefficient (Surface #4 is −12.68; Surface #6 is −4.32).

**Figure 7:**
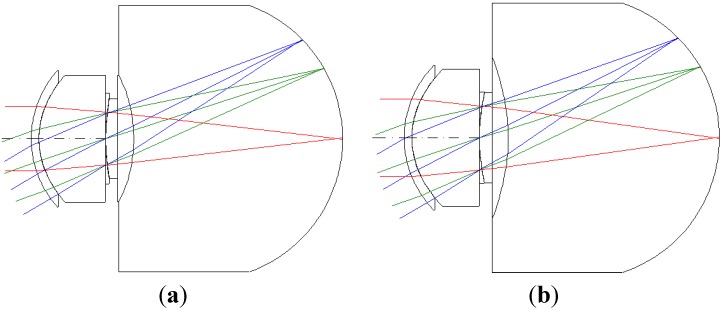
The 2D layout after applying the built-in optimization method in CODE V. (**a**) 5 mm; (**b**) 6 mm.

**Figure 8:**
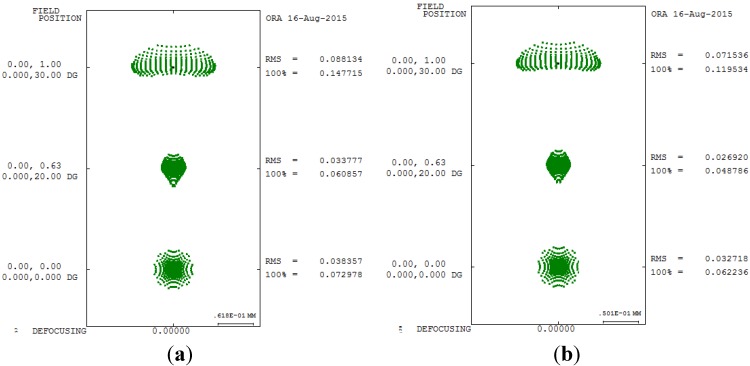
Spot size diagram after applying the built-in optimization method in CODE V. (**a**) 5 mm; (**b**) 6 mm.

**Figure 9:**
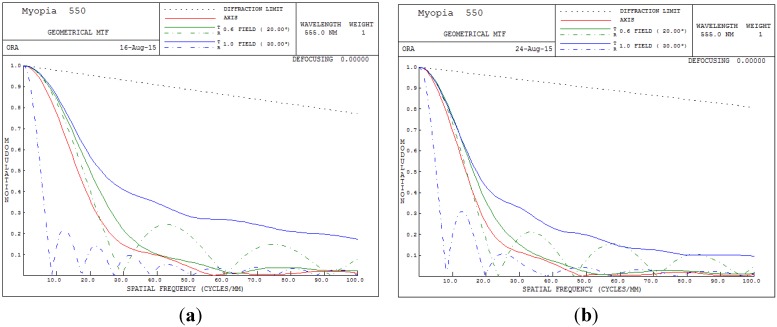
MTF plot after applying the built-in optimization method in CODE V. (**a**) 5 mm; (**b**) 6 mm.

The third-order aberration results after correcting the 550 degrees myopia and 175 degrees astigmatism by using the built-in optimization method in CODE V is reported in Table 8. Figure 7 shows a 2D layout obtained after applying the built-in optimization method. Figure 7 shows the imperfect focusing points on a retina. Figure 8 and Figure 9 show the spot size diagram and MTF performance after applying the built-in optimization method, respectively. In Figure 8, the spot size plot is denser than in Figure 5 for the imperfect human eye state, implying that the built-in optimization method reduced aberrations. Moreover, the MTF values also increased as shown in Figure 9, indicating clearer human eye vision.

**Table 8:** Third-order aberrations after applying the built-in optimization method in CODE V.

	Aberration	SA	TCO	RMS
Pupil Size				
5 mm		0.01	−0.0162	0.032718
6 mm		0.01	−0.01781	0.038357

### 3.2. Principle of Genetic Algorithm

In Table 3 and Figure 9, we can see that the built-in optimization of SA yielded good results; however, the optimization method for the TCO, RMS and MTF do not perform very well. Hence, a GA was used to overcome this problem and achieve a balanced optimization for the TCO, RMS and MTF performance.

The concept of GA is derived from Darwin’s concept of evolution, that is, the theory of natural selection, which postulates survival of the fittest. The less fit are eliminated because the reproduction process, the resilience of individual organisms is relatively easy to find a good mate with each other to reproduce the next generation through after a long evolution, and finally will make the organism can adapt to the environment given to stable growth conditions. Some scholars have observed such changes in order to adapt to the environment and natural phenomena, simulate biological evolution between children raised on the best application engineering [19,20,21].

The GA technique is applied to the optical design of the artificial IOL. The advantages of integrating the GA optimization process with the optical design are the obtention of the overall physical artificial crystal focusing function and higher flexibility. Use this way to match and change data, that achieving superior outcomes to those of the CODE V built-in function. Simulation results showed that not only was the state of the SA reduced but the TCO, MTF, and spot size also improved effectively, leading to the accurate focusing of light on the retina. However, the third-order aberration of SA worsens, because the optical design always involves a tradeoff between all aberrations. The IOL design mainly involves designing the curvature and thickness to improve the vision quality. Therefore, the chromosomes were the curvature and thickness, which randomly generated a new curvature and thickness gene according to the ethnic population. A calculated process used the fitness function to determine the good vision qualities for facilitating the subsequent crossover and mutation mechanisms. The fitness function used in the proposal is defined as:

(5) $$fin\left(i\right)=\sum_{z=1}^{1}w_{1}\left|{\text{SA}}_{Z}\right|+w_{2}\left|{\text{TCO}}_{Z}\right|\text{ for }i\;=1,2,\ldots ,\;pop\_size$$

where $w_{1}$ and $w_{2}$ are the weights, *pop_size* is the population size and the roulette wheel method is applied to the selection operation. The whole area of the wheel is one. Each lens system based on the fitness probability is assigned an area in the wheel, that is:

*fit*_roulette_(*i*) = (*fit*_max_ + *fit*_min_) – *fit*(*i*) for *i* = 1,2,..., *pop_size* (6)

where the *fit*_max_ and *fit*_min_ are the maximum and minimum values of *fin*(*i*).

According to Equation (6), the fitness probability of the lens system chosen to implement crossover operation is:

(7) $$q\left(i\right)\;=\;{fit}_{\text{roulette}}\left(i\right)/\sum_{a=1}^{pop\_size}fit_{\text{roulette}}\left(a\right)\text{ for }i\;=\;1,2,...,\;pop\_size$$

and:

*q*(0) = 0 (8)

the cumulative probability of the wheel is:

(9) $$v\left(i\right)\;=\;\sum_{j=1}^{i}q\left(j\right)/\sum_{a=1}^{pop\_size}p\left(n\right)\text{ for }i\;=\;1,2,...,\;pop\_size$$

A random number α between 0 and 1 is generated for running the selection operation. We would select the genes of the ith lens system if *q*(*i* − 1) < α ≤ *q*(*i*). Two genes parameters of lens systems are selected to implement the crossover operation. Let the two selected genes parameters be *X* = (*x*_1_, *x*_2_,…, *x*_2_) and *Y* = (*y*_1_, *y*_2_,…, *y*_2_). The offspring of these two parents is the sequence *Z* = (*z*_1_, *z*_2_,…, *z*_2_) and its crossover genes are defined by:

(10) $$z_{i}=\beta x_{i}+\left(1-\beta \right)y_{i}$$

where β is a random number between 0 and 1. Following the selection and crossover operations, the better genes would be the ones that propagate the next generation lens system. However, the creatures in the real world evolve their genes to fit their environment better. A mutation probability *p*_m_ is defined and a random number between 0 and 1 is generated in the GA process. The mutation operation is implemented when α > *p*_m_. The mutation operation used in the proposal is defined as:

(11) $$z_{i}=z_{i}\pm \beta \Delta$$

where Δ is a constraint to prevent the result from being divergent. β is a random number between 0 and 1. The GA process is to implement the selection, crossover and mutation operations. The DLS method is added to the GA process to enhance the optimization result.

The merit function of current optical software focuses on reducing the image spot size, making high image resolution or an attractive MTF result. The other weightings from *w*_1_ to *w*_2_ are assigned the values all of 1. The population size, generation, crossover rate, and mutation rate are set to 100, 70, 0.8 and 0.2 respectively in running the optimization.

**Figure 10:**
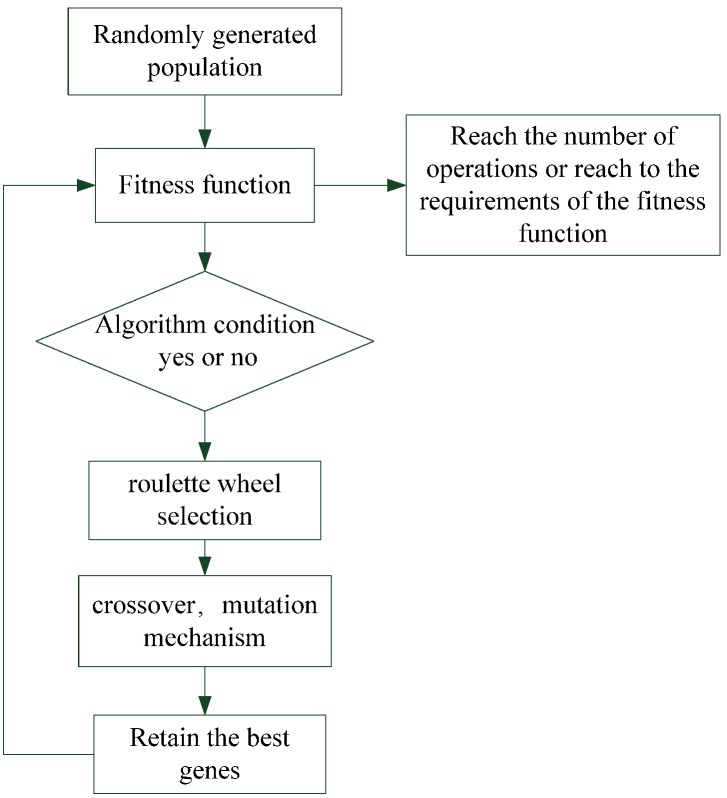
Flowchart of the genetic algorithm (GA) used in the intraocular lens (IOL) design.

Finally, after crossover and mutation, the resulting chromosomes’ parameters are substituted into CODE V for designing an IOL. The flow chart of the GA is shown in Figure 10.

**Figure 11:**
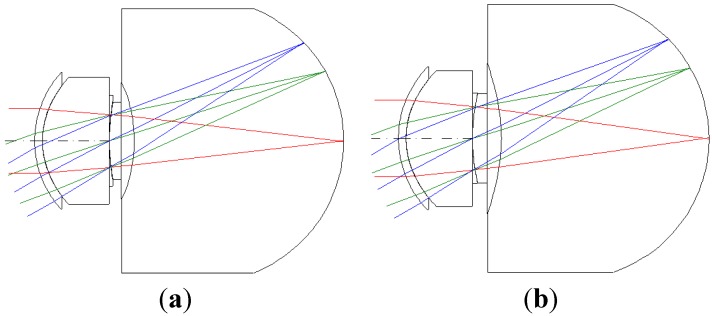
The 2D diagram after applying the GA optimization method. (**a**) 5 mm; (**b**) 6 mm.

The 2D layout and third-order aberrations obtained after correcting the vision for 550 degrees myopia and 175 degrees astigmatism by using the proposed GA optimization method are as presented in Figure 11 and Table 9. Comparing the built-in optimization of CODE V (Figure 7) and the proposed GA optimization method (Figure 11), we can see that the proposed GA method provides a better focusing point on the retina.

**Table 9:** Third-order aberrations after applying the GA optimization method.

	Aberration	SA	TCO	RMS
Pupil Size				
5 mm		0.032994	−0.011763	0.020633
6 mm		0.014618	−0.010201	0.031816

The improvement rate “*D*” of the built-in optimization method and the proposed GA method can be calculated using:

(12) D=[(|X|−|Y|)|Y|]×100%

where *Y* is the third-order aberration value after applying the built-in optimization method, and *X* is the third-order aberration after applying the proposed GA optimization method. 

**Figure 12:**
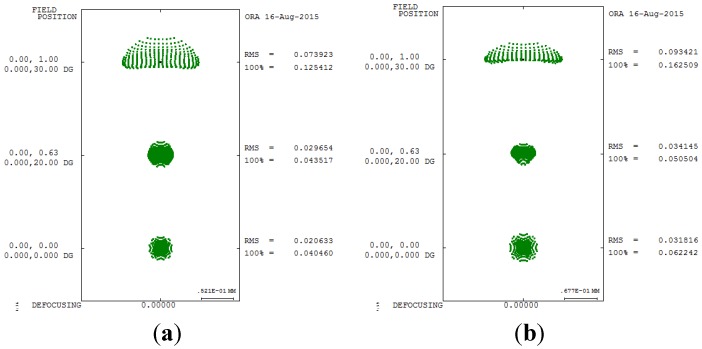
Spot size diagram after applying the GA optimization method. (**a**) 5 mm; (**b**) 6 mm.

**Figure 13:**
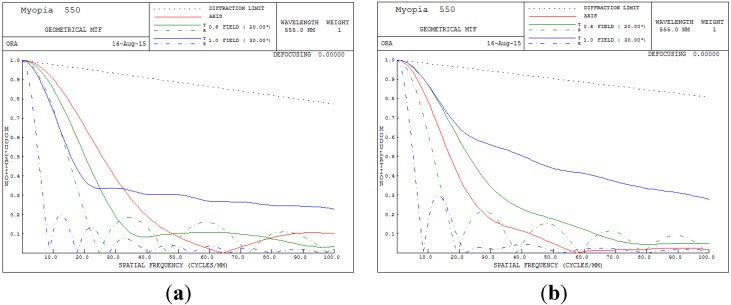
MTF plot obtained after applying the GA optimization method. (**a**) 5 mm; (**b**) 6 mm.

Table 10 and Table 11 presents the improvement rate proposed GA method of third-order aberrations for the 550 degrees myopia and 175 degrees astigmatism states for different pupil sizes. A comparison of the spot diagrams in Figure 8 and Figure 12 shows a smaller spot size in the proposed GA method, implying that the proposed GA method yields better geometry for aberrations. A comparison of Figure 9 and Figure 13 shows higher MTF curves for the proposed GA method, confirming that a higher vision resolution can be achieved using the proposed GA optimization method.

**Table 10:** Improvement rate of third-order aberrations after applying the proposed GA optimization method for pupil size 5 mm.

Quality Characteristics	SA	TCO
550 degrees myopia and 175 degrees astigmatism condition	−0.073302	−0.052464
GA optimization	0.032994	−0.011763
Difference values	0.040308	0.040701
Improvement rate (%)	53.98%	77.57%

**Table 11:** Improvement rate of third-order aberrations after applying the proposed GA optimization method for pupil size 6 mm.

Quality Characteristics	SA	TCO
550 degrees myopia and 175 degrees astigmatism condition	−0.126666	−0.075548
GA optimization	0.014618	−0.010201
Difference values	0.112048	0.065347
Improvement rate (%)	88.45%	86.49%

Table 12 and Table 13 list the improvement rates of the built-in and proposed optimization methods for 550 degrees myopia and 175 degrees astigmatism. Although the SA correction by the proposed optimization method is worse than that by the built-in optimization method, the crucial aberrations, TCO and RMS, in the human eye for pupil size 6 mm are improved by approximately 43% and 17%, respectively.

From optical design perspectives, MTF values represent the entire image contrast and sharpness and have a contrast range of 0 to 1. Generally, images cannot be analyzed after optical design when the MTF value is nearly 0. To determine the ideal image quality of the human eye vision, the MTF must be higher than 0.5 when the spatial frequency is 6, and the MTF must be approximately higher than 0.3 when the spatial frequency is 15.

**Table 12:** Comparisons of third-order aberrations of the built-in and proposed optimization methods for 550 degrees myopia and 175 degrees astigmatism for pupil size 5 mm.

Quality Characteristics	SA	TCO	RMS
CODE V optimization	0.01	−0.016200	0.032718
GA optimization	0.032994	−0.011763	0.020633
Difference values	0.022	0.004437	0.012
Improvement rate (%)	−229%	27.38%	36.93%

**Table 13:** Comparisons of third-order aberrations of the CODE V built-in and proposed optimization methods for 550 degrees myopia and 175 degrees astigmatism for pupil size 6 mm.

Quality Characteristics	SA	TCO	RMS
CODE V optimization	0.01	−0.01781	0.038357
GA optimization	0.014618	−0.010201	0.031816
Difference values	0.004618	0.007609	0.006541
Improvement rate (%)	−46.18%	42.72%	17.05%

Figure 6, Figure 9 and Figure 13 show MTF plots for the different myopic and astigmatic states for different pupil sizes. Figure 6 plots the myopia and astigmatism conditions of the human eye, and the MTF values at different spatial frequencies do not exceed 0.1; hence, human vision becomes unacceptable. Figure 9b shows the MTF plot after applying the built-in optimization method for pupil size 6 mm; the MTF value is 0.695 when the spatial frequency is 10. However, when the spatial frequency reaches 30, the MTF value rapidly drops below 0.115, implying that the image quality of the human eye worsens. In conclusion, the built-in method in CODE V is workable and satisfies all MTF conditions. Figure 13b shows the MTF plot for the proposed GA optimization method for pupil size 6 mm; the proposed MTF values are 0.783 and 0.177 when the spatial frequencies are 10 and 30 cycles/degree, respectively, implying that the proposed GA design method is workable and really improves human vision.

Table 14 and Table 15 show the MTF values and improvement rate for different simulation conditions and at spatial frequencies of 10, 20 and 30 lp/mm.

**Table 14:** Modulation transfer function (MTF) values for pupil size 5 mm.

Optimal State	10 lp/mm	20 lp/mm	30 lp/mm
Myopic and astigmatism	0.003	0.001	0
Internal CODE V	0.768	0.356	0.145
GA	0.901	0.657	0.389
Improvement rate(%)	14.76%	45.81%	62.72%

**Table 15:** MTF values for pupil size 6 mm.

Optimal State	10 lp/mm	20 lp/mm	30 lp/mm
Myopic and astigmatism	0.005	0	0.007
Internal CODE V	0.695	0.253	0.115
GA	0.783	0.391	0.177
Improvement rate(%)	11.23%	35.29%	35.02%

## 4. Conclusions

The main conclusion of this study is that the optical design (*i.e.*, designing an IOL for improving SA, TCO, MTF, and spot size for 550 degrees myopia and 175 degree astigmatic states) of an IOL by using the GA optimization method is advantageous. Compared with the built-in optimization method in CODE V, the improvement rate achieved using the proposed GA scheme was lower than 46% for spherical aberration but increased 43% for TCO and 17% for RMS of pupil size 6 mm condition. Although the improvement of the SA aberration was less than that in the CODE V built-in method, the crucial aberrations, TCO and RMS, were both substantially higher. According to the MTF plot, compared with the built-in optimization method, the proposed GA optimization method improves by nearly 15% and 46%at spatial frequencies of 10 and 20 for pupil size 5 mm, and 11% and 35% at spatial frequencies of 10 and 20 for pupil size 6 mm, respectively. Even at a spatial frequency of 30, the proposed GA method outperforms the traditional built-in optimization method by 63% for pupil size 5 mm, and 35% for pupil size 6 mm. In conclusion, the simulation results of third-order aberrations, spot size, and MTF performance show that the proposed GA method for aspherical lens design can be effectively used in artificial IOL design.
